# Colorectal cancer incidence and survival inequalities among labour immigrants in Belgium during 2004–2013

**DOI:** 10.1038/s41598-022-19322-1

**Published:** 2022-09-21

**Authors:** Katrien Vanthomme, Michael Rosskamp, Harlinde De Schutter, Hadewijch Vandenheede

**Affiliations:** 1 Sociology Department, Interface Demography, Vrije Universiteit Brussel, Pleinlaan 2, 1050 Brussels, Belgium; 2 Department of Public Health and Primary Care, Faculty of Medicine and Health Sciences, Ghent University, C. Heymanslaan 10, 9000 Ghent, Belgium; 3 Research Department, Belgian Cancer Registry, Koningsstraat 215, 1210 Brussels, Belgium

**Keywords:** Cancer epidemiology, Colorectal cancer, Cancer epidemiology, Colorectal cancer, Cancer epidemiology

## Abstract

Colorectal cancer (CRC) is one of the leading causes of cancer-related morbidity and mortality. We aim to map out differences in CRC incidence and survival between first-generation traditional labour immigrants of Italian, Turkish and Moroccan descent and native Belgians; and assess the contribution of socioeconomic position (SEP) to these differences. Individually-linked data of the 2001 Belgian Census, the Crossroads Bank for Social Security and the Belgian Cancer Registry are used. Age-standardized incidence rates and incidence rate ratios are calculated by country of origin, with and without adjusting for SEP. For CRC patients, 5-year relative survival rates and the relative excess risk for dying within five years after diagnosis are calculated by migrant origin. Lower CRC incidence was observed among immigrants compared to native Belgians, in particular among non-Western immigrants, which could not be explained by SEP. Survival inequalities were less clear, yet, after adjusting for age and stage at diagnosis and educational attainment, we observed a survival advantage among Turkish and Italian immigrant men. Health gains can be made for the native population by adapting lifestyle. The later stage at diagnosis for immigrants is of concern. Barriers regarding screening as perceived by the vulnerable groups should be identified.

## Introduction

Colorectal cancer (CRC) is one of the leading causes of cancer-related morbidity and mortality^1^. In Belgium, 7990 people got diagnosed with CRC in 2019^2^, and 2754 people died from CRC in 2016^3^. Belgium scores high with an age-standardized incidence rate (ASR) of 134.9 per 100,000 Person-Years (PY) among the population aged 50–74 years in 2020, compared with the world average of 72.2 per 100,000 PY^4^. In contrast, the age-standardized mortality rate among Belgians aged 50–74 years (33.3 per 100,000 PY) was close to the world average (29.3 per 100,000 PY) in 2020.

CRC develops from precancerous polyps^5^. The occurrence of CRC is associated with lifestyle factors such as nutrition, physical activity and obesity^6^. The prognosis of the disease depends, amongst others, on the tumour stage at diagnosis which can be improved by early detection^7,8^. Hence, early detection can reduce CRC incidence and mortality. In Belgium, organized screening was introduced in 2009 for the Walloon and Brussels Region and 2013 for the Flemish Region among the population aged 50–74 years^7,9^.

Immigrants make up an important share of the European populations^10^, which had led to a growing interest in research on the health of immigrants. Belgium is a particularly suitable setting to analyse immigrant differences in CRC given its long history of immigration^11^. In the 1950s and 1960s, Belgium was in strong need of labour immigrants to overcome the labour shortages in the heavy industries^11^. Particularly, large groups of Southern European, Turkish and Moroccan men immigrated, later followed by their wives. Nowadays, these first-generation (FG) immigrants have reached older ages, making it important to follow-up their health status and health care issues^12^. Studying CRC by immigrant origin may (1) identify important health inequalities; and (2) reveal important clues on disease aetiology and hence prevention^12–14^ as FG immigrants have changed environment throughout their life course.

Previous research in Belgium already assessed CRC mortality patterns by immigrant origin^15,16^. For the traditional labour immigrant groups in Belgium, mainly from Turkey, Morocco and Italy, mortality patterns were generally advantageous compared with native Belgians, although with variations by immigrant origin and gender. A CRC mortality advantage was particularly observed among immigrants from Turkish and Moroccan descent but much less among immigrants from Italian descent. This migrant mortality advantage (MMA) (at least for Turkish and Moroccan immigrants) compared to the native population is often referred to as the mortality paradox as immigrants tend to have a more disadvantaged socioeconomic position (SEP) compared to the native population^17^. Such a MMA generally occurs for lifestyle-related diseases and cancers, such as CRC, but is less present or even reversed for infectious-related pathologies^10,12,14,18–22^. In addition, the MMA tends to wear off with length of stay in the country of destination due to acculturation to the lifestyle of the host country^10,12,18,20^.

What is less known however, is whether the observed MMA may be explained by differences in disease occurrence and/or survival^23^. Therefore, this study aims to assess differences in CRC occurrence and survival between the native and the FG immigrant population in Belgium. Previous research already looked into CRC incidence patterns by immigrant origin and length of stay (without accounting for stage at diagnosis), generally showing lower CRC incidence among Turkish and Moroccan immigrants^24^. Moreover, an increase in CRC incidence was observed among Turkish and Moroccan male immigrants with a length of stay of more than 30 years. In contrast, CRC incidence of Italian immigrants did not differ from incidence patterns of native Belgians. To our knowledge, CRC survival patterns by immigrant origin have not been studied in Belgium. As survival is strongly related to stage at diagnosis, we might expect lower survival among immigrant groups given their lower participation in cancer screening programs^7,25–27^, which is related to both their migrant background and their lower SEP. A Dutch study however did not observe significant differences in CRC survival by migrant background^13^.

With this study, we want to contribute to the current knowledge on CRC differences in three ways. *First*, by mapping out differences in CRC incidence and stage at diagnosis by immigrant origin and gender using data at the Belgian population level. *Second*, by studying survival inequalities by immigrant origin and gender. *Third*, by assessing the contribution of SEP to these immigrant differences in CRC incidence and relative survival. As immigrants are likely to be situated in lower SEP^8,17^, it is important to account for SEP when assessing incidence and survival differences as this may partially explain observed associations.

## Data and methods

We used individually-linked data from three different data sources: the Belgian census of October 1st 2001 containing sociodemographic and socioeconomic information (i.e. immigrant origin, year of immigration, civil status, region of residence, educational attainment and home ownership); the Belgian Cancer Registry containing all CRC diagnoses in Belgian residents between January 1st 2004 and December 31st 2013 as well as stage and time of diagnosis; and the Crossroads Bank for Social Security providing emigration and vital status until July 1st 2017. These three data sources were individually-linked through a trusted third party (e-Health) and provided to us in a pseudonymized way. In this study, we focused on FG immigrants of Italian, Turkish and Moroccan origin aged 50–74 years at the start of the follow-up and compared them with native Belgians within the same age range. We focused on FG immigrants as they have been exposed to both the home and host country’s environment. The age group was chosen because CRC is very common in this age group^2^ and as it is the age range covered by the population-based screening programs.

The outcome variables in this study are (1) being diagnosed with CRC during the follow-up period 2004–2013; and (2) among diagnosed patients 5-year relative survival (RS). Only malignant primary tumours were included as cases. We used the 10th International Classification of Diseases (ICD-10) to include all primary invasive cancer diagnosis of colon (C18-C19) and rectum (C20)^28^. All tumours, whether histologically confirmed and/or clinically diagnosed have been included. If patients had multiple CRC tumours within the study period, only the first occurring tumour was taken into account. The stage at diagnosis was based on the clinical and pathological Tumour-lymph Nodes-Metastasis (TNM) staging system: 6th edition for 2004–2009^29^ and 7th edition for 2010–2013^30^. The combined TNM stage prioritized pathological over clinical stage except in case of clinical distant metastases which were always designated stage IV^31^. Invasive cancers that, after neo-adjuvant treatment (chemo- and/or radiation therapy), could not be detected anymore when biopsy/surgery of the primary tumour was undertaken (post-operative pathological examination) were registered as stage 0. Since the number of CRC cases were rather limited in the migrant groups, we grouped this variable into three categories: stages 0, I and II (early stage); stages III and IV (late stage) and ‘unknown’ stage.

The main explanatory variable of interest was immigrant origin. This variable was constructed using a stepwise method based on the country of origin of individuals and their parents, thereby maximizing the proportion of the population with migrant roots. If individuals could be linked to their parents at the census of 2001 and the nationality at birth of one of the parents was non-Belgian, then this nationality was used to define individuals’ country of origin. If individuals could not be linked to their parents or parents’ origin was missing in 2001, we checked whether this information was available in the census of 1991 and repeated the first step. If individuals could not be linked to their parents, or if both parents had Belgian origin, we used individuals’ nationality at birth to define country of origin. If this information was also lacking, current nationality was used to define country of origin. For this study, we selected only FG immigrants of Italian, Turkish and Moroccan descent, as these were the main traditional labour immigrant groups in Belgium. Consequently, we included only Italian, Turkish and Moroccan immigrants who migrated themselves to Belgium (i.e. FG immigrants) and excluded those who immigrated only recently to Belgium (in 1991 or later).

Next to immigrant origin, several sociodemographic and socioeconomic variables of interest were taken into account. The first one was ‘civil status’ which consists of being married, single, divorced or widowed. This indicator serves as a proxy of social support which may be related to CRC screening^7,32,33^. ‘Region of residence’ at census was also included and comprises the Flemish, Brussels-Capital and the Walloon Region. This variable was included as screening policy is organized at the regional level in Belgium. Additionally, we included two indicators of SEP as they may represent different forms of disadvantage during different periods of life^23^. ‘Educational attainment’ reflects chances early in life and may therefore not be the most suitable indicator among immigrants, yet it is an important indicator of ‘knowledge’ and may therefore be important for health literacy and screening participation^34^. Educational attainment was categorized according to the International Standard Classification of Education (ISCED): primary education or no diploma (ISCED 0-1), lower secondary education (ISCED 2), upper secondary education (ISCED 3-4), and tertiary education (ISCED 5-6). We also included ‘home ownership’, which refers to the economic assets at the household level. Home ownership differentiates between tenants and owners of a dwelling. We did not impute the missing values on the socioeconomic variables but included them as separate category as we do not consider them to be random.

In the first part of the analyses, we aimed to assess whether FG immigrants have different CRC incidence patterns and stage at diagnosis compared to native Belgians. To do so we calculated both absolute and relative CRC incidence inequalities. We estimated truncated ASR and 95% confidence intervals (C.I.) by country of origin for immigrants and native Belgians. The person-time at risk by 5-year age groups was calculated for each person in the study cohort between 2004 and 2013 to calculate age-specific incidence rates. The incidence rates were directly standardized using the European standard population as a reference^35^. In addition, we calculated relative incidence rate ratios (IRR) and 95% C.I. by immigrant origin using a Poisson distribution with the log of the person-time as the offset variable. The IRRs were adjusted for age at the start of the follow-up. To assess the impact of all other variables of interest, they were added separately to the additional models: civil status (model 2); educational attainment (model 3); home ownership (model 4); and region at time of census (model 5).

The second part of the analyses concerns RS inequalities. This analysis was performed for patients diagnosed with CRC between 2004 and 2013, with vital status provided until July 1st 2017. We calculated 5-year RS as a proxy for CRC-specific survival, hereby excluding the effect of different background mortality in population groups^36^. RS was calculated as the ratio of the observed survival in a group of patients to the expected survival in a comparable group from the general population. This expected survival was based on sex-, age-, region- and calendar-year-specific national life-tables provided by Statistics Belgium^37^ using the Ederer II method^38^. To compare RS of CRC patients with and without a migrant origin, we calculated the relative excess risk (RER) and 95% C.I. for dying within five years after diagnosis using Poisson regression models^39^. These models represent the ratio of the excess hazard of dying due to CRC during the first five years after diagnosis for a particular migrant group compared to the native Belgian reference group. To assess the impact of all the variables of interest, we assessed different models adding age, combined TNM stage at diagnosis and sociodemographic and socioeconomic variables resulting in the following models: adjustment for age at time of diagnosis (Model 1); age at time of diagnosis and combined TNM stage at diagnosis (Model 2); age at time of diagnosis, combined TNM stage at diagnosis and civil status (Model 3); age at time of diagnosis, combined TNM stage at diagnosis and educational attainment (Model 4); age at time of diagnosis, combined TNM stage at diagnosis and home ownership (Model 5); and age at time of diagnosis, combined TNM stage at diagnosis and region at census (Model 6). All analyses have been performed using SAS 9.3 (SAS Institute Inc.).

### Ethical standards

This research as well as the data adhere to the ethical code of scientific research in Belgium, see: https://www.belspo.be/belspo/organisation/publ/pub_ostc/Eth_code/ethcode_en.pdf.

All authors have signed the ethical code. The project was approved by the Medical ethics committee of UZ Brussel, VUB-B.U.N. 43201734363. The study is in accordance with relevant guidelines and regulations.

## Results

### Description of the study population

The study population contained all native Belgian and FG Italian, Turkish and Moroccan immigrant men and women aged 50–74 years at the start of the follow-up, as described in Table 1. The latter two groups were on average a bit younger than native Belgians and Italian immigrants and had lived less years in Belgium compared with Italian immigrants. The majority of Italian immigrants lived in Wallonia, while Turkish immigrants tended to live more often in Flanders and Moroccan immigrants in the Brussels Capital Region. Among native Belgians, about three-quarters were married, and this percentage was even larger among the immigrant groups. While about 20% of native Belgians were highly educated, this percentage was much lower among the immigrant groups. Importantly, information on education was lacking for a large proportion of the immigrants. Finally, more than three-quarters of native Belgians and Italian immigrants were owners of a dwelling, whereas this percentage was smaller among Turkish and Moroccan immigrants.

**Table 1:** Description of the study population aged 50 to 74 years at the start of the follow-up (January 1st 2004), by gender and migrant origin, Belgium.

	Men				Women			
	Belgian	Italian	Turkish	Moroccan	Belgian	Italian	Turkish	Moroccan
Number of persons	1,128,706	33,811	6786	14,953	1,212,655	31,051	5885	10,235
Mean age at start follow-up	60.64	60.23	59.81	60.00	61.15	61.18	59.35	58.47
Mean years since immigration	–	34.80	28.07	29.22	–	35.49	27.06	27.23
**Region (%)**								
Flanders	65.21	12.82	48.19	30.33	63.41	9.33	48.14	26.84
Brussels Capital Region	5.42	10.22	27.48	52.53	6.30	10.19	28.58	57.37
Wallonia	29.37	76.95	24.33	17.14	30.30	80.47	23.28	15.79
**Civil status (%)**								
Married	78.79	84.00	95.99	94.21	71.37	73.06	81.94	77.84
Single	7.52	5.21	0.87	1.41	5.01	2.57	0.25	0.48
Divorced	10.10	7.93	1.97	3.67	10.63	6.51	2.04	8.21
Widow	3.59	2.86	1.16	0.71	12.99	17.86	15.77	13.47
**Educational attainment (%)**								
Primary or no diploma	21.34	30.87	35.81	14.15	24.72	34.83	21.21	9.80
Lower secondary	26.45	21.95	8.97	11.70	28.80	19.55	4.27	6.00
Upper secondary	20.14	11.37	5.13	7.61	18.61	8.34	2.75	2.77
Tertiary	20.37	5.18	3.82	3.18	15.59	2.79	1.29	0.73
Missing	11.70	30.64	46.27	63.35	12.29	34.48	70.48	80.70
**Home ownership (%)**								
Owner	79.76	78.75	64.68	55.90	78.30	78.17	61.99	53.61
Tenant	16.24	16.53	25.98	35.68	17.59	17.37	28.50	38.12
Missing	4.00	4.72	9.34	8.43	4.11	4.46	9.52	8.27

### Description of the colorectal cancers and absolute incidence inequalities by migrant origin in Belgium

Table 2 shows the characteristics of newly diagnosed CRC cases. Moroccan immigrants were generally a bit younger than native Belgians at time of CRC diagnosis. Among Turkish immigrant men, the percentage with unknown stage at diagnosis was highest compared with all other groups. Turkish and Moroccan immigrant women were diagnosed in later stages than native Belgian women.

**Table 2:** Description of colorectal cancer cases among the study population aged 50 to 74 years, by gender and migrant origin, Belgium, 2004–2013.

	Men				Women			
	Belgian	Italian	Turkish	Moroccan	Belgian	Italian	Turkish	Moroccan
Total colorectal cancer cases	19,154	534	64	127	12,800	300	35	62
Mean age at diagnosis	66.60	66.13	67.07	65.31	66.62	66.22	65.03	63.22
**Combined TNM stage (%)**								
Early stage (0-II)	45.86	45.88	42.19	42.52	44.33	48.34	34.28	37.10
Late stage (III-IV)	44.97	42.13	37.51	48.03	46.59	43.33	62.85	56.45
Missing	9.16	11.99	20.31	9.45	9.09	8.33	2.86	6.45

The truncated ASR of CRC was highest among native Belgian men and women: 188.1 new CRC cases per 100,000 men per year (95% C.I.: 185.3–190.9) and 115.1 per 100,000 women per year (95% C.I.: 113.0–117.2) (Table 3). Compared with native Belgians, CRC incidence rate was lower among Turkish and Moroccan immigrant men and women, whereas no difference was observed with Italian immigrants. For instance, the CRC incidence rate for Moroccan immigrant men was less than half the one of native Belgian men, with a rate amounting to 88.1 per 100,000 men per year (95% C.I.: 72.4–103.8). As in men, CRC incidence rates of Turkish and Moroccan immigrant women were much lower than that of native Belgian women, with rates of 69.1 per 100,000 (95% C.I.: 44.3–93.9) and 66.1 per 100,000 women per year, respectively (95% C.I.: 48.9–83.3).

**Table 3:** Number of cases, person-years, truncated age-standardized incidence rates (ASR per 100,000 person-years including 95% Confidence intervals [95% C.I.]), number of deaths among cases and 5-year relative survival (RS including 95% C.I.) among the study population aged 50 to 74 years, by gender and migrant origin, Belgium, 2004–2013.

	Incidence			Survival	
	Number of cases	Person-years	Truncated ASR (95% C.I.)	Number of deaths	5-year RS (95% C.I.)
**Men**					
Belgian	19,154	9,014,118	188.09 (185.29–190.88)	7599	66.9 (66.1–67.7)
Italian	534	276,954	171.07 (155.61–186.54)	211	65.2 (54.7–74.4)
Turkish	64	54,894	104.14 (77.97–130.30)	< 20	83.2 (68.9–93.6)
Moroccan	127	124,557	88.06 (72.35–103.77)	52	68.1 (63.1–72.8)
**Women**					
Belgian	12,800	9,673,596	115.10 (112.96–117.24)	4385	69.6 (68.7–70.5)
Italian	300	251,346	109.58 (95.19–123.98)	95	65.4 (51.3–76.8)
Turkish	35	50,026	69.10 (44.34–93.85)	< 20	81.3 (62.2–93.0)
Moroccan	62	89,943	66.08 (48.85–83.31)	23	73.0 (66.9–78.3)

Because of confidentiality issues, the exact number of cases are not reported when below 20.

### Relative colorectal cancer incidence inequalities by migrant origin in Belgium

Table 4 shows that Turkish and Moroccan immigrant men had respectively 43% (IRR: 0.57; 95% C.I.: 0.44–0.73) and 51% (IRR: 0.49; 95% C.I.: 0.41–0.58) lower CRC incidence rates compared with native Belgian men. Similar results were observed among women: Turkish and Moroccan immigrant women had 43% (IRR: 0.57; 95% C.I.: 0.41–0.80) and 42% (IRR: 0.58; 95% C.I.: 0.45–0.75) lower CRC incidence compared with native Belgian women. Immigrants from Italian descent had lower CRC incidence rates compared with native Belgians, yet less pronounced (IRR_men_: 0.93; 95% C.I.: 0.85–1.01; IRR_women_: 0.90; 95% C.I.: 0.80–1.00). Adjusting the models for sociodemographic and socioeconomic variables (Table 4, models 2–5) did not alter the association between immigrant origin and CRC incidence among men and women.

**Table 4:** Relative colorectal cancer incidence inequalities among the study population aged 50 to 74 years by gender and migrant origin, adjusted for age and sociodemographic and socioeconomic indicators, Belgium, 2004–2013.

Relative incidence rate ratios (and 95% Confidence Intervals)					
Men	Model 1	Model 2	Model 3	Model 4	Model 5
**Migrant origin**					
Belgian	(ref.)	(ref.)	(ref.)	(ref.)	(ref.)
Italian	0.93 (0.85–1.01)	0.93 (0.85–1.01)	0.92 (0.84–1.00)	0.93 (0.85–1.01)	0.99 (0.90–1.08)
Turkish	**0.57 (0.44–0.73)**	**0.57 (0.45–0.73)**	**0.56 (0.44–0.72)**	**0.56 (0.44–0.72)**	**0.57 (0.45–0.73)**
Moroccan	**0.49 (0.41–0.58)**	**0.49 (0.41–0.58)**	**0.47 (0.40–0.56)**	**0.48 (0.40–0.57)**	**0.49 (0.41–0.59)**
Age at start	**1.07 (1.07–1.07)**	**1.07 (1.07–1.08)**	**1.07 (1.07–1.07)**	**1.07 (1.07–1.08)**	**1.07 (1.07–1.07)**
**Civil status**					
Married		(ref.)			
Single		1.01 (0.95–1.06)			
Divorced		**1.05 (1.00–1.10)**			
Widow		1.05 (0.97–1.13)			
**Educational**					
Primary or no diploma			**1.04 (1.00–1.09)**		
Lower secondary			**1.08 (1.04–1.13)**		
Upper secondary			**1.08 (1.03–1.13)**		
Tertiary			(ref.)		
Missing			**1.10 (1.05–1.16)**		
**Home ownership**					
Owner				(ref.)	
Tenant				**1.08 (1.04–1.12)**	
Missing				1.03 (0.96–1.11)	
**Region at census**					
Flanders					(ref.)
Brussel Capital Region					0.95 (0.89–1.01)
Wallonia					**0.89 (0.86–0.95)**

Women	Model 1	Model 2	Model 3	Model 4	Model 5
**Migrant origin**					
Belgian	(ref.)	(ref.)	(ref.)	(ref.)	(ref.)
Italian	0.90 (0.80–1.00)	0.90 (0.80–1.01)	0.90 (0.80–1.01)	0.90 (0.80–1.00)	0.93 (0.83–1.05)
Turkish	**0.57 (0.41–0.80)**	**0.57 (0.41–0.80)**	**0.59 (0.42–0.82)**	**0.57 (0.41–0.79)**	**0.57 (0.41–0.80)**
Moroccan	**0.58 (0.45–0.75)**	**0.58 (0.46–0.75)**	**0.60 (0.47–0.78)**	**0.58 (0.45–0.74)**	**0.59 (0.46–0.76)**
Age at start	**1.06 (1.06–1.06)**	**1.06 (1.06–1.06)**	**1.06 (1.06–1.06)**	**1.06 (1.06–1.06)**	**1.06 (1.06–1.06)**
**Civil status**					
Married		(ref.)			
Single		**1.11 (1.03–1.20)**			
Divorced		0.96 (0.90–1.02)			
Widow		1.00 (0.95–1.06)			
**Educational**					
Primary or no diploma			1.04 (0.98–1.10)		
Lower secondary			**1.05 (1.00–1.11)**		
Upper secondary			1.03 (0.97–1.10)		
Tertiary			(ref.)		
Missing			0.98 (0.92–1.05)		
**Home ownership**					
Owner				(ref.)	
Tenant				1.02 (0.98–1.07)	
Missing				1.05 (0.96–1.14)	
**Region at census**					
Flanders					(ref.)
Brussel Capital Region					0.96 (0.90–1.03)
Wallonia					**0.93 (0.90–0.97)**

Model 1 is adjusted for age at the start of the follow-up; Model 2 is adjusted for age at the start of the follow-up and civil status; Model 3 is adjusted for age at the start of the follow-up and educational attainment; Model 4 is adjusted for age at the start of the follow-up and home ownership; Model 5 is adjusted for age at the start of the follow-up and region at time of census. Results significant at the *p* < 0.05-level are in Bold.

### CRC survival inequalities by migrant origin in Belgium

For most groups, 5-year relative survival amounted to about 65–70% (Table 3). Yet, among Turkish immigrant men, 5-year RS was significantly higher (83%) compared with native Belgian men, although we must be cautious due to small numbers.

We observed no differences in the RER of dying after being diagnosed with CRC by immigrant origin in the age-adjusted model (Table 5, model 1). However, after adjusting for stage at diagnosis and educational attainment, both Turkish and Italian immigrant men showed a survival advantage compared to native Belgian men (Table 5, model 4) with an excess risk of dying among Turkish CRC patients of 54% lower than native Belgian CRC patients (RER: 0.46; 95% C.I.: 0.24–0.88). The excess risk of dying of Italian immigrant CRC patients was 16% lower compared to native Belgian patients (RER: 0.84; 95% C.I.: 0.70–1.00). In addition, after adjusting for stage at diagnosis and region, Turkish immigrant men showed a survival advantage compared to native Belgian men (Table 5, model 6) with an excess risk of dying of 48% lower than native Belgian CRC patients (RER: 0.52; 95% C.I.: 0.27–0.99). Among women, no such differences were observed.

**Table 5:** Relative 5-year colorectal cancer survival inequalities among the study population aged 50 to 74 years by gender and migrant origin, adjusted for age at time of diagnosis, combined TNM stage and sociodemographic and socioeconomic indicators, Belgium, diagnosed between 2004–2013 and followed until 2017.

Relative excess risk (RER) of dying within five years after diagnosis (and 95% Confidence Intervals)						
Men	Model 1	Model 2	Model 3	Model 4	Model 5	Model 6
**Migrant origin**						
Belgian	(ref.)	(ref.)	(ref.)	(ref.)	(ref.)	(ref.)
Italian	0.92 (0.76–1.10)	0.92 (0.77–1.10)	0.92 (0.77–1.10)	**0.84 (0.70–1.00)**	0.90 (0.76–1.08)	0.88 (0.73–1.05)
Turkish	0.54 (0.27–1.08)	0.54 (0.28–1.02)	0.59 (0.31–1.11)	**0.46 (0.24–0.88)**	0.53 (0.28–1.01)	**0.52 (0.27–0.99)**
Moroccan	1.03 (0.73–1.45)	1.06 (0.77–1.47)	1.13 (0.82–1.56)	0.91 (0.66–1.27)	0.96 (0.69–1.33)	0.94 (0.68–1.32)
Age at diagnosis	**1.01 (1.01–1.02)**	**1.02 (1.01–1.02)**	**1.02 (1.02–1.03)**	**1.01 (1.01–1.02)**	**1.02 (1.02–1.03)**	**1.02 (1.01–1.02)**
**Combined TNM stage**						
Early stage (0-II)		(ref.)	(ref.)	(ref.)	(ref.)	(ref.)
Late stage (III-IV)		**6.23 (5.68–6.84)**	**6.16 (5.62–6.76)**	**6.22 (5.67–6.83)**	**6.16 (5.62–6.76)**	**6.26 (5.70–6.88)**
Missing		**6.00 (5.36–6.73)**	**5.94 (5.30–6.65)**	**5.99 (5.34–6.71)**	**5.96 (5.32–6.67)**	**5.99 (5.34–6.72)**
**Civil status**						
Married			(ref.)			
Single			**1.68 (1.53–1.84)**			
Divorced			**1.36 (1.24–1.48)**			
Widow			**1.32 (1.15–1.51)**			
**Educational attainment**						
Primary or no diploma				(ref.)		
Lower secondary				**1.39 (1.27–1.52)**		
Upper secondary				**1.19 (1.09–1.30)**		
Tertiary				**1.16 (1.06–1.28)**		
Missing				**1.57 (1.42–1.74)**		
**Home ownership**						
Owner					(ref.)	
Tenant					**1.47 (1.37–1.57)**	
Missing					**1.64 (1.45–1.85)**	
**Region at census**						
Flanders						(ref.)
Brussels Capital Region						**1.27 (1.13–1.42)**
Wallonia						1.06 (0.99–1.13)

Women	Model 1	Model 2	Model 3	Model 4	Model 5	Model 6
**Migrant origin**						
Belgian	(ref.)	(ref.)	(ref.)	(ref.)	(ref.)	(ref.)
Italian	0.85 (0.66–1.09)	0.89 (0.69–1.13)	0.89 (0.70–1.14)	0.83 (0.65–1.07)	0.89 (0.70–1.14)	0.85 (0.66–1.09)
Turkish	0.63 (0.27–1.45)	0.60 (0.27–1.31)	0.61 (0.28–1.33)	0.53 (0.24–1.17)	0.61 (0.28–1.32)	0.57 (0.26–1.25)
Moroccan	1.27 (0.81–2.01)	1.14 (0.72–1.80)	1.16 (0.73–1.83)	1.01 (0.64–1.60)	1.05 (0.67–1.66)	1.07 (0.68–1.69)
Age at diagnosis	**1.01 (1.01–1.02)**	**1.02 (1.01–1.03)**	**1.02 (1.01–1.02)**	**1.02 (1.01–1.02)**	**1.02 (1.01–1.03)**	**1.02 (1.01–1.03)**
**Combined TNM stage**						
Early stage (0-II)		(ref.)	(ref.)	(ref.)	(ref.)	(ref.)
Late stage (III-IV)		**7.45 (6.59–8.41)**	**7.43 (6.58–8.39)**	**7.38 (6.54–8.33)**	**7.36 (6.52–8.30)**	**7.45 (6.59–8.41)**
Missing		**6.35 (5.47–7.38)**	**6.33 (5.45–7.35)**	**6.31 (5.44–7.33)**	**6.33 (5.45–7.34)**	**6.32 (5.44–7.34)**
**Civil status**						
Married			(ref.)			
Single			**1.44 (1.25–1.65)**			
Divorced			**1.17 (1.05–1.32)**			
Widow			**1.25 (1.13–1.38)**			
**Educational attainment**						
Primary or no diploma				(ref.)		
Lower secondary				**1.32 (1.17–1.48)**		
Upper secondary				**1.14 (1.01–1.28)**		
Tertiary				**1.17 (1.03–1.33)**		
Missing				**1.35 (1.18–1.55)**		
**Home ownership**						
Owner					(ref.)	
Tenant					**1.33 (1.22–1.44)**	
Missing					**1.31 (1.12–1.53)**	
**Region at census**						
Flanders						(ref.)
Brussels Capital Region						1.15 (1.00–1.33)
Wallonia						**1.08 (1.00–1.17)**

Model 1 is adjusted for age at time of diagnosis; Model 2 is adjusted for age at time of diagnosis and combined TNM stage at diagnosis; Model 3 is adjusted for age at time of diagnosis, combined TNM stage at diagnosis and civil status; Model 4 is adjusted for age at time of diagnosis, combined TNM stage at diagnosis and educational attainment; Model 5 is adjusted for age at time of diagnosis, combined TNM stage at diagnosis and home ownership; Model 6 is adjusted for age at time of diagnosis, combined TNM stage at diagnosis and region at time of census. Results significant at the *p* < 0.05-level are in Bold.

## Discussion and conclusion

This study is the first in Belgium to map out differentials in CRC incidence and survival by immigrant origin at a population scale and the extent to which these disparities are related to socioeconomic deprivation, civil status, region of residence and stage at diagnosis. To do so, we used nation-wide individually-linked data containing CRC diagnoses during a 10-year observation period, based on cancer registration information covering more than 95% of the Belgian population, together with detailed tumour information^3^, and vital status up to five years after diagnosis as well as socioeconomic and sociodemographic information for the entire study population aged 50 to 74 years. This rich dataset enabled us to calculate precise CRC incidence rates and 5-year relative survival by immigrant origin and gender. Immigrants are a very heterogeneous group: exposures in the home country, acculturation, mastering the host country’s language, cultural and religious beliefs are all aspects that are related to health and that can differ by country of origin^8^. Hence the importance of assessing incidence and relative survival rates for various groups of immigrant origin separately. In this study we chose to include three groups of traditional labour immigrants in Belgium to be compared with native Belgians. We chose these groups as they make up the largest traditional labour immigrant groups reaching older ages in Belgium, and as they have been exposed a significant amount of time to both home and host country’s environment. Nevertheless, the number of CRC cases in the immigrant groups, especially among Turkish and Moroccan immigrants was rather low, hence caution is needed when interpreting the results.

The extent to which the observed CRC patterns may be associated with missing or incorrect remigration patterns (salmon bias) is unknown but we assume this bias to be minor as previous research proved for mortality figures^17^. Given the nature of this bias, we may however assume that it might be more relevant for the survival figures and less for the incidence patterns^12^.

The data enabled us to also assess the contribution of sociodemographic and socioeconomic variables to CRC differences by immigrant origin. It is crucial to assess the contributions of immigration, SEP and sociodemographic factors as they all are likely to be associated with the disease^40^.

Another merit of the dataset is the long follow-up of the cancer registry which allowed us to perform detailed analyses by immigrant origin and gender. Such national, comprehensive cancer registries are not available in many countries, let alone with the availability of linked socioeconomic, sociodemographic and mortality data. We also disposed of stage at diagnosis, which is an important marker of disease prognosis. Yet, this stage at diagnosis was more likely to be unknown among male immigrants and conversely less likely to be unknown among female immigrants, a topic that deserves further study.

Still, with this dataset we were not able to consider differentials in health care utilization nor lifestyle factors. Documenting disparities in certain health behaviours (e.g. nutritional diet, physical activity, body mass index or alcohol and tobacco consumption), CRC screening or treatment could have enhanced the knowledge on the origin of the observed differences in CRC incidence and survival. Due to low numbers we were also not able to calculate combined TNM stage-specific incidence and relative survival rates, but we were able to account for stage at diagnosis in the relative survival models. In addition, comparison with incidence patterns of the home country was not possible due to the lack of nationwide cancer registries in these countries where cancer registries operate more as regional initiatives^41,42^. Finally, as indicators of SEP, we decided to include educational attainment as a proxy of cultural capital and home ownership as a proxy of economic capital and civil status as sociodemographic characteristic. Yet, these variables were measured at the 2001 census and changes over time could not be taken into account. Moreover, given the age range of the study population we decided not to include employment status as the majority of the population would have reached retirement age. Finally, we did not dispose of information on income, which could be an important variable regarding health care access and lifestyle.

This study revealed some important clues on CRC incidence and survival patterns within the Belgian immigrant population. *First*, we observed lower CRC incidence rates among FG immigrants as compared to native Belgians. This finding is in line with previous research in the Netherlands^13,20^, Norway^43^ and Germany^14^. The advantage was most pronounced among non-Western immigrants from Turkish and Moroccan descent and almost negligible among immigrants from Italian descent, which corresponds to the observation that cancer incidence varies between populations from low- and middle-income countries versus high-income countries^14,43^. CRC incidence figures from the countries of origin for the same age group 50–74 years from the Globocan website reflect the same pattern: CRC incidence in 2020 was highest in Belgium, followed by Italy and much lower in Turkey, and Morocco ^4^. This observation may be explained by differences in lifestyle^13,43–46^. CRC incidence is very much related to a Western lifestyle, including a diet containing high intake of meat, fat and total calories, as well as high levels of obesity, smoking and heavy alcohol consumption^47^. As opposed to that, immigrants from Turkish and Moroccan descent combine the favourable Mediterranean nutritional pattern containing a high uptake of fruit and vegetables with low levels of alcohol consumption^45,46,48^. On the contrary, obesity and physical inactivity may be more prevalent among non-western groups^22,49,50^. These findings were corroborated with data from the Belgian Health Interview Survey (BHIS)^51^. The BHIS data show that Moroccan immigrants had much lower levels of male smokers than the other groups, whereas in women this applied to Turkish immigrant women too. Concerning alcohol overconsumption, Turkish and Moroccan immigrants did have lower prevalence compared to native Belgians and Italian immigrants. The same Western-non-Western divide was observed for fruit and vegetables uptake.

*Second*, the differences in CRC incidence in immigrants could not be explained by sociodemographic or socioeconomic differences. Although CRC incidence was higher among unmarried persons, low-educated people, tenants (only in men) and people living in Flanders, accounting for these variables did not alter the observed CRC incidence differences in immigrants versus native Belgians. This suggests that differences in lifestyle are more important to explain the incidence pattern and that lifestyle is more strongly related to immigrant origin than to SEP. Apart from immigrant origin, we did observe sociodemographic and socioeconomic factors to be associated with CRC incidence. These patterns were as expected, with higher CRC incidence rates among the most vulnerable groups. For instance, persons without a partner were more often diagnosed with CRC compared with persons with a partner, which might be related to a less healthy diet or a less active lifestyle^52^.

*Third*, survival inequalities were less outspoken, especially in comparison with incidence inequalities. In the relative models however, we observed a survival advantage among Turkish and Italian immigrant men when accounting for stage at diagnosis and educational attainment. This suggests that both the later stage at diagnosis of immigrants and their generally lower educational attainment might be negatively related to their survival chances. Studies on cancer survival among immigrants remain rather scarce. Yet a study in Australia observed lower mortality among CRC patients with an immigrant origin^53^, and a study in the Netherlands observed a slightly better relative survival among CRC patients with an immigrant background as opposed to native Dutch patients^13^. As noted above, we cannot entirely exclude possible selective return migration among those immigrants diagnosed with CRC, that could bias the survival estimates^54^. Yet, we deem this unlikely as health services and treatment are better in the host country and as most immigrants have their families present in Belgium^55^.

*Finally*, an important finding was that although immigrants had lower CRC incidence, Turkish immigrant women and Moroccan immigrants were diagnosed at later stages, as was observed in Germany as well^8^. The difference in stage at diagnosis may be due to differences in CRC screening uptake^26,49,56,57^ and may have implications for their overall prognosis. Data from the BHIS indeed show a lower screening attendance for CRC among Turkish and Moroccan immigrants compared with native Belgians and Italian immigrants^51^, which was also observed among immigrant groups in the Netherlands^25^. Previous research has shown that immigrants perceive several access barriers to health care utilization and to screening programs in particular^14,25,27,49,57,58^. Perceived barriers are e.g. low health literacy, language barriers, a lack of supporting social network, insecurities about health encounters, stigma and fear. This study was conducted among FG labour immigrants, who often only have a limited command of Belgium’s national languages, which may hamper their timely use of appropriate health care^8,25,59^. Moreover, previous research showed that immigrants were less familiar with someone with CRC, and therefore may be less likely to acquire information or to participate in screening programs^58^. Immigrants were also more likely to perceive the impact of a CRC diagnosis on their families as a burden, and they received less often appropriate recommendations on screening programs of their doctor, as doctors themselves still have the perception of CRC to be a western disease. In the Region of Flanders, a lower uptake in the organized CRC screening program was also observed for people with a migration background, but also for men and people situated in the lower socioeconomic strata^7^. Yet, given the fact that this study includes cancer diagnoses between 2004 and 2013, and the fact that organised screening programs were introduced in 2009 for the Walloon and Brussels Region and 2013 for the Flemish Region, there must be other factors that play a role in explaining differences in incidence and stage at diagnosis.

The observation of lower CRC incidence among the group of FG labour immigrants in Belgium suggests that significant health gains can be made especially for the native population by adapting certain lifestyle habits such as a healthy nutritional diet, lower alcohol and smoking consumption and being more physically active. Future research, however, should also assess CRC incidence patterns among second-generation immigrants to verify whether the health advantage still exist within this group. It is known that second-generation immigrants are more likely to have adapted their lifestyle to that of the host population^60^, and hence are at a higher risk of developing CRC. Previous research in Belgium that took length of stay in Belgium into account, provided support for a cancer transition with increased CRC incidence by longer duration of stay in Belgium, at least among men^24^. This might also apply to second-generation immigrants who were born here. This, together with the observation that immigrants of Turkish or Moroccan descent were diagnosed at later stages is of concern. Screening programs can create inequalities if not all strata of the population are reached equally^7,27^. Bearing in mind that the immigrant population is now reaching older ages, it is essential to reach a high cancer screening coverage within the immigrant population as well^7,49,57^. General practitioners could play an important role in promoting participation in screening programs, as immigrants are less aware of CRC, have less health literacy and perceive several barriers^58,61^. Another issue that need to be studied in the future is the time trend in CRC incidence differences as well as young- versus non-young-onset CRC incidence patterns, which has been shown to vary by migrant origin in Germany^62^. Furthermore, identifying the actual barriers as perceived by the vulnerable groups in society that hamper the appropriate use of health care is essential^33^. As it is crucial to assess the contributions of migration, SEP and sociodemographic factors in health and mortality, effective cancer control policies should consequently also focus on the interaction of these factors. Finally, this study was made possible by performing a direct linkage between different data sources. It would be valuable for future studies to provide other interesting data linkages that could enhance the knowledge on e.g. differences in risk factors patterns or health care utilization by specific subgroups.

## Data Availability

Data are from a census-cancer registry-linked mortality follow-up study and cannot be made available due to privacy issues. These data are from administrative nature, hence no informed consent was needed. Researchers can gain full access to the data by submitting an application to the Belgian Data Protection Authority (DPA). In order to get permission to use data from the Belgian population register linked to census data an authorization request (in Dutch or French) needs to be submitted to the Belgian DPA. The authorization request includes an application form and additional forms regarding data security. The necessary forms for the authorization request can be downloaded from the Belgian DPA website (www.dataprotectionauthority.be). Next to information on the applicant and the list of requested data, the authorization request should further specify which data are requested (e.g. aggregated or pseudonymized), why the data from the population register are necessary, for which time span data will be stored, how and where these data will be stored, and who will have access to the data.
